# Positive and negative survivor-specific psychosocial consequences of childhood cancer: the DCCSS-LATER 2 psycho-oncology study

**DOI:** 10.1007/s11764-023-01394-1

**Published:** 2023-05-11

**Authors:** Anne Maas, Heleen Maurice-Stam, Alied M. van der Aa-van Delden, Elvira C. van Dalen, Eline van Dulmen-den Broeder, Wim J. E. Tissing, Jacqueline J. Loonen, Helena J. H. van der Pal, Andrica C. H. de Vries, Marry M. van den Heuvel-Eibrink, Geert O. Janssens, Cécile Ronckers, Sebastian Neggers, Dorine Bresters, Marloes Louwerens, Birgitta A. B. Versluys, Margriet van der Heiden-van der Loo, Leontien C. M. Kremer, Marloes van Gorp, Martha A. Grootenhuis, on behalf of the Dutch LATER study group

**Affiliations:** 1 Princess Máxima Center for Pediatric Oncology, Utrecht, The Netherlands; 2 Amsterdam UMC/Location VUmc, Amsterdam, The Netherlands; 3 Beatrix Children’s Hospital, University of Groningen/University Medical Center Groningen, Groningen, The Netherlands; 4 Radboud University Medical Center, Nijmegen, The Netherlands; 5 Sophia Children’s Hospital, Erasmus Medical Center, Rotterdam, The Netherlands; 6 Department of Radiation Oncology, University Medical Center Utrecht, Utrecht, The Netherlands; 7 Division of Childhood Cancer Epidemiology, Institute of Medical Biostatistics Informatics and Epidemiology, University Medical Center of the Johannes Gutenberg University, Mainz, Germany; 8 Department of Medicine, Section Endocrinology, Erasmus Medical Center, Rotterdam, The Netherlands; 9 Willem Alexander Children’s Hospital, Leiden University Medical Center, Leiden, The Netherlands; 10 Leiden University Medical Center, Leiden, The Netherlands; 11 Wilhelmina Children’s Hospital/University Medical Center Utrecht, Utrecht, The Netherlands; 12 Department of Pediatrics, Amsterdam UMC, University of Amsterdam, Amsterdam, The Netherlands

**Keywords:** *Childhood cancer survivors*, *Psychosocial*, *Quality of life*, *Impact of cancer*, *Long-term survivorship*

## Abstract

**Purpose:**

Numerous studies investigated generic psychosocial outcomes in survivors of childhood cancer (CCS). The present study aimed to describe survivor-specific psychosocial consequences in CCS, and to identify socio-demographic and medical associated factors.

**Methods:**

CCS from the Dutch Childhood Cancer Survivor Study (DCCSS)-LATER cohort (diagnosed 1963–2001) part 2 (age ≥ 18 years, diagnosed < 18 years, ≥ 5 years since diagnosis) completed the Benefit & Burden Scale (BBSC) and the Impact of Cancer–Childhood Cancer (IOC-CS). Items were scored on a 5-point Likert scale (range 1–5). We examined outcomes with descriptive statistics, and socio-demographic and medical associated factors with regression analyses, corrected for multiple testing (*p *< 0.004).

**Results:**

CCS, *N* = 1713, age mean (M) 36 years, 49% female, ≥ 15 years since diagnosis, participated. On average, CCS reported ‘somewhat’ Benefit (M = 2.9), and ‘not at all’ to ‘a little’ Burden (M = 1.5) of childhood cancer. Average scores on IOC-CS’ positive impact scales ranged from 2.5 (Personal Growth) to 4.1 (Socializing), and on the negative impact scales from 1.4 (Financial Problems) to 2.4 (Thinking/Memory). Apart from cognitive problems, CCS reported challenges as worries about relationship status, fertility, and how cancer had affected siblings. Female sex was associated with more Personal Growth, and more negative impact. CCS more highly educated, partnered, and employed had higher positive and lower negative impact. CCS older at diagnosis reported more positive impact. CNS tumor survivors and those who had head/cranium radiotherapy had higher negative impact. CNS tumor survivors reported less positive impact.

**Conclusion and implications:**

The majority of CCS reported positive impact of cancer while most CCS reported little negative impact. While this may indicate resiliency in most CCS, health care providers should be aware that they can also experience survivor-specific challenges that warrant monitoring/screening, information provision and psychosocial support.

**Supplementary Information:**

The online version contains supplementary material available at 10.1007/s11764-023-01394-1.

## Introduction

Over the past decades childhood cancer survival rates have risen [1]. The population of survivors of childhood cancer (CCS) has increased, sparking interest among researchers to study long-term psychosocial adaptation in CCS. Numerous studies have examined general psychosocial outcomes in survivors, such as health related quality of life, anxiety, distress, and depression [2–6]. Recently, we examined generic psychosocial outcomes in a Dutch nationwide cohort study [7]. The results were in line with previous studies, showing that the majority of CCS are resilient and score within normal ranges on generic, standardized measures of psychosocial functioning. However, these generic measures on which most studies have relied may lack specificity in exploring how cancer affects CCS. First, most studies have focused on negative psychosocial impact of childhood cancer, while CCS may also experience positive consequences of their illness [8–10]. Second, studies looking at issues of particular concern to survivors, i.e. survivor-specific outcomes, are currently scarce [11, 12], and most are limited to a relatively small sample size or a specific group of CCS such as children [13], young adults [14] or survivors of acute lymphoblastic leukemia [15].

CCS can experience both long-term benefit as well as burden. Although benefit has not yet been studied in a large cohort, studies including this concept have found that the majority of CCS (≥ 85%) report at least one positive outcome from their cancer experience [16–19]. Benefits such as obtaining priorities about what is important in life, having greater appreciation for the value of life, and experiencing a stronger bond with family and friends were reported. At the same time, CCS can experience negative impact of their illness, such as fear of recurrence, fertility concerns and worries regarding late effects [11, 12, 20–22]. A study among adolescent and young adult cancer survivors found that most (> 88%) reported moderate cancer-related worry in at least one area, of which worry about future health was the most common concern [23]. Positive and negative impact of cancer do not fall on opposite ends of a continuum, but can coexist in the same person [13, 24].

Zebrack et al. [11, 12] distinguish several domains in which CCS can experience positive or negative impact of childhood cancer. Studies taking these domains into account [11, 14, 15] found that CCS reported higher positive than negative impact. This may reflect either the growth that CCS experience or a coping strategy in which positive impact is emphasized and negative impact denied. Perceived positive and negative impact of childhood cancer are associated with psychosocial well-being [11, 14], and may therefore provide a promising target for interventions.

Although few studies have looked at associated factors, several characteristics appeared to be associated with positive and negative impact. Positive impact was found to be lower and negative impact higher among CCS who were unemployed, not married/partnered, and of lower income [12]. More negative impact was found among CCS with lower education [12], and among female CCS [15], while female CCS did experience greater positive impact in terms of Personal Growth and Socializing [25]. Regarding cancer-related medical characteristics, CCS appeared less likely to report positive and negative impact as they move further in time from diagnosis [12, 13]. CCS older at diagnosis experienced higher benefit [13, 15], while CCS of central nervous system (CNS) tumors experienced higher burden [13, 25]. Inconsistent results were found for those who had experienced cancer recurrence [13, 15].

Looking beyond generic psychosocial outcomes and taking the survivor-specific impact of cancer into consideration, both positive and negative, is essential to fully understand psychosocial functioning in long-term CCS and to provide targeted interventions. This study is the first to look at survivor-specific constructs in a large, nationwide cohort. First, we aimed to describe survivor-specific psychosocial consequences of childhood cancer (mean scores and frequencies), including perceived positive and negative impact. Second, we aimed to identify socio-demographic and cancer-related medical factors associated with survivor-specific psychosocial consequences.

## Methods

### Design and population

This study is part of the Dutch Childhood Cancer Survivor Study (DCCSS)-LATER cohort part 2; clinical visit and questionnaire study (DCCSS-LATER 2 study). Details of the cohort are described elsewhere [26]. The DCCSS-LATER 2 study is a cross-sectional, nationwide cohort study consisting of all CCS diagnosed between 1963 and 2001, aged < 18 years at diagnosis, ≥ 5 years since diagnosis at time of study, and treated in one of the seven former Dutch pediatric oncology centers. The medical ethics board of all seven centers approved the study protocol.

The DCCSS-LATER 2 study included a sub-study on psychosocial outcomes (the LATER Psycho-oncology study). For this part of the study, CCS aged ≥ 18 years were eligible. CCS who gave informed consent for the LATER Psycho-oncology study received a questionnaire addressing psychosocial functioning including measures assessing survivor-specific outcomes. The questionnaire was provided by mail or at CCS’ visit to the outpatient clinic for the DCCSS-LATER 2 study, and completed from home.

### Measures

#### Benefit and Burden Scale Children: BBSC

The BBSC measures benefit finding and disease-related burden [27]. With permission of the developer, we have made a minimal adjustment to the formulation of one item to make it suitable for adults. It consists of two scales addressing perceived Benefit (10 items, Cronbach’s α 0.90) and Burden (10 items, Cronbach’s α 0.87) of childhood cancer. Items were scored on a 5-point Likert scale ranging from ‘Not at all true for me’ (1) to ‘Very much true for me’ (5). Higher scores indicate higher benefit and burden. The BBSC has good psychometric properties [13, 27, 28]. The BBSC was translated into Dutch through a forward–backward translation process. The author of the original BBSC crosschecked the backward translation for any inconsistencies.

#### Impact of Cancer – Childhood Survivor: IOC-CS

The IOC-CS measures perceived positive and negative impact of childhood cancer in life domains particularly relevant to CCS [11]. It consists of five positive impact scales with 3–8 items (Socializing, Cronbach’s α 0.69; Talking with Parents, Cronbach’s α 0.92; Body & Health, Cronbach’s α 0.82; Health Literacy, Cronbach’s α 0.72; Personal Growth, Cronbach’s α 0.68), and six negative impact scales with 2–12 items (Thinking/Memory problems, Cronbach’s α 0.73; Sibling Concerns, Cronbach’s α 0.58; Life Challenges, Cronbach’s α 0.84; Relationship Concerns, Cronbach’s α 0.51 for partnered CCS, and Cronbach’s α 0.71 for non-partnered CCS; Financial Problems, Cronbach’s α 0.80), and some separate items. Items were scored on a 5-point Likert scale ranging from ‘Not at all’ (1) to ‘Very much’ (5). Higher scores indicate respectively more positive and negative impact. The IOC-CS has good psychometric properties [25]. The IOC-CS was translated into Dutch through a forward–backward translation process. The author of the original IOC-CS crosschecked the backward translation for any inconsistencies.

#### Socio-demographic and medical characteristics

The socio-demographic characteristics sex, education level (low: primary education, lower vocational education, lower and middle general secondary education; middle: middle vocational education, higher general secondary education, pre-university education; high: higher vocational education, university), having a partner (yes/no), and being employed (yes/no) were obtained via questionnaires in the DCCSS-LATER 2 study in the same period as the outcome measures were assessed. Attained age (birth month and year) was obtained from the DCCSS-LATER registry.

The following medical characteristics were obtained from the DCCSS-LATER registry: diagnosis, age at diagnosis, treatment, time since diagnosis and cancer recurrence. As radiotherapy was previously identified as a predictor of worse psychosocial outcomes [4, 29, 30], we studied radiotherapy in more detail by addressing different regions of exposure (head/cranium, spinal, total body irradiation, thorax, abdominal/pelvic, testes, neck, upper extremities, lower extremities, radioisotopes).

### Statistical analysis

Independent t-tests and Chi-Square tests were used to test for differences between participants and non-participants on socio-demographic and medical characteristics, with Cohen’s d and Cramer’s V as effect sizes. Survivor-specific psychosocial outcomes were examined with descriptive statistics: means, mean item scale scores, and percentages. If at least half of the items of a scale were completed, item scores were imputed with the mean item score of the concerning scale.

To distinguish between CCS in the degree of experienced impact of childhood cancer and to enhance the interpretability of the results, we rescaled the 5-point Likert scales (BBSC and IOC-CS) into three categories: not at all or a little bit (little impact: 1, 2) somewhat (some impact: 3), and quite a bit or very much (much impact: 4, 5). We analyzed these categories with descriptive statistics (frequencies).

Associations of socio-demographic and medical factors with the outcomes were assessed with multiple linear regression analyses. We used the continuous scale scores as outcomes to make full use of the variance in the data. Prior to the analysis basic assumptions were checked. Listwise deletion was applied to the regression analyses, given the small proportion of missing data and the large sample size. Diagnosis and treatment were analyzed in separate regression models, as these characteristics are interdependent. Following Cohen [31] for mean differences between two groups and for regression coefficients of dichotomous independent variables, we considered effect sizes of 0.2, 0.5. and 0.8 respectively small, moderate and large. For regression coefficients of continuous independent variables and for Cramer’s V, 0.1, 0.3 and 0.5 were considered small, moderate and large, respectively. Very small effect sizes (Cohen’s d < 0.2 or Cramer’s V < 0.1) are not considered relevant, and these results are therefore not discussed. A significance level of 0.004 was used for the regression analyses to correct for multiple testing; 0.05 divided by the number of 12 scales.

## Results

### Participants

**Fig. 1:**
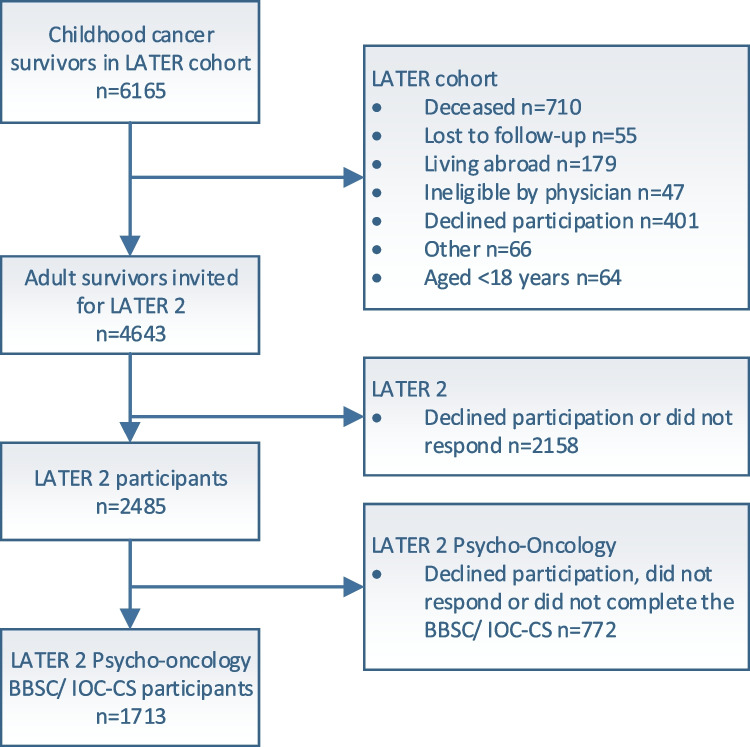
Flowchart of participants

The childhood cancer survivor LATER cohort consisted of 6165 CCS of which 5455 were alive (Fig. 1). A total of 4643 adult CCS were invited for the DCCSS-LATER 2 study and 2485 participated (53.5%). Of these 2485 CCS, 1713 (68.9%) completed the BBSC and/or the IOC-CS. Participants were compared to non-participants who gave permission to use their data (2238/2930). Participating CCS had a mean (M) age of 36.0 years (SD 9.3, range 18.3–70.9), 48.9% were female and mean time since diagnosis was 29.2 years (SD 8.5, range 15.3–55.0). Some small (V ≤ 0.1) differences were found between participants and non-participants on some socio-demographic and medical characteristics (Table 1).

**Table 1:** Socio-demographic and Medical Characteristics CCS: Participants Versus Non-participants

	Participants (*N* = 1713)			Non-participants (*N* = 2238)			Cohen’s *d*
	*M*	*SD*	Range	*M*	*SD*	Range	
Age at study (years)	35.97	9.32	18.29—70.88	35.18	9.27	18.02—70.52	.08**
Age at first diagnosis (years)	6.78	4.73	0.00 – 17.96	6.61	4.71	0.00 – 17.95	.04
Time since first diagnosis (years)	29.19	8.49	15.34 – 55.01	28.57	8.24	15.44 – 56.18	.07*
	*% (N)*			*% (N)*			Cramer’s *V*
Socio-demographic characteristics							
Sex							.09***
Male	51.1 (875)			59.5 (1341)			
Female	48.9 (838)			40.1 (897)			
Partnered	78.8 (1210)						
Educational attainment							
Low	13.0 (220)						
Middle	41.9 (710)						
High	45.1 (765)						
Employed	85.0 (1441)						
Medical characteristics							
Age at first diagnosis (years)							.02
0–5	52.9 (907)			53.9 (1206)			
6–11	27.8 (476)			28.4 (635)			
12–17	19.3 (330)			17.7 (397)			
Time since first diagnosis (years)							.05
10–19	15.6 (267)			17.1 (383)			
20–29	41.2 (706)			42.9 (961)			
30–39	31.1 (533)			29.0 (648)			
40–49	10.6 (182)			10.3 (230)			
50–59	1.5 (25)			0.7 (16)			
Recurrence (yes)	13.3 (227)			11.9 (266)			.02
Diagnosis							
Leukaemia	34.4 (589)			33.9 (759)			.01
Lymphoma	19.3 (331)			18.8 (420)			.01
CNS tumor	9.2 (158)			11.6 (260)			.04*
Neuroblastoma	5.9 (101)			4.9 (109)			.02
Retinoblastoma	0.4 (7)			0.8 (17)			.02
Renal tumour	11.4 (195)			11.0 (246)			.01
Hepatic tumour	1.0 (17)			1.1 (24)			.00
Bone tumour	6.4 (109)			4.9 (110)			.03*
Soft-tissue sarcoma	7.2 (124)			7.5 (168)			.01
Germ cell tumour	3.1 (53)			4.2 (95)			.03
Other tumor	1.5 (26)			1.3 (30)			.01
Unspecified tumour	0.2 (3)			0 (0)			.03*
Treatment							
Surgery (yes)	50.4 (860)			51.2 (1140)			.01
Chemotherapy (yes)	87.5 (1499)			81.5 (1822)			.08***
Radiotherapy (yes)	39.3 (673)			30.9 (691)			09***
Radiotherapy regions							
Head/ Cranium	18.6 (318)			16.3 (363)			.03
Spinal	5.0 (86)			4.0 (90)			.02
Total body irradiation	3.9 (66)			2.3 (52)			.04**
Thorax	6.8 (116)			4.9 (110)			.04*
Pelvic area	8.4 (144)			6.8 (151)			.03
Testes	0.5 (9)			0.4 (9)			.01
Neck	4.0 (69)			3.3 (73)			.02
Upper extremities	0.7 (12)			0.7 (16)			.00
Lower extremities	1.3 (23)			1.1 (25)			.01
Radioisotopes	1.1 (19)			0.7 (15)			.02

^*^p-value < 0.05, **p-value < 0.01, ***p-value < 0.001, significant differences (*p*< .05) are presented in bold. Because of missing values, *N* varies slightly across variables. Data were missing for non-participating survivors who declined the use of their data in the DCCSS-LATER registry (*n*= 692)

### Positive and negative impact of childhood cancer

#### BBSC

**Fig. 2:**
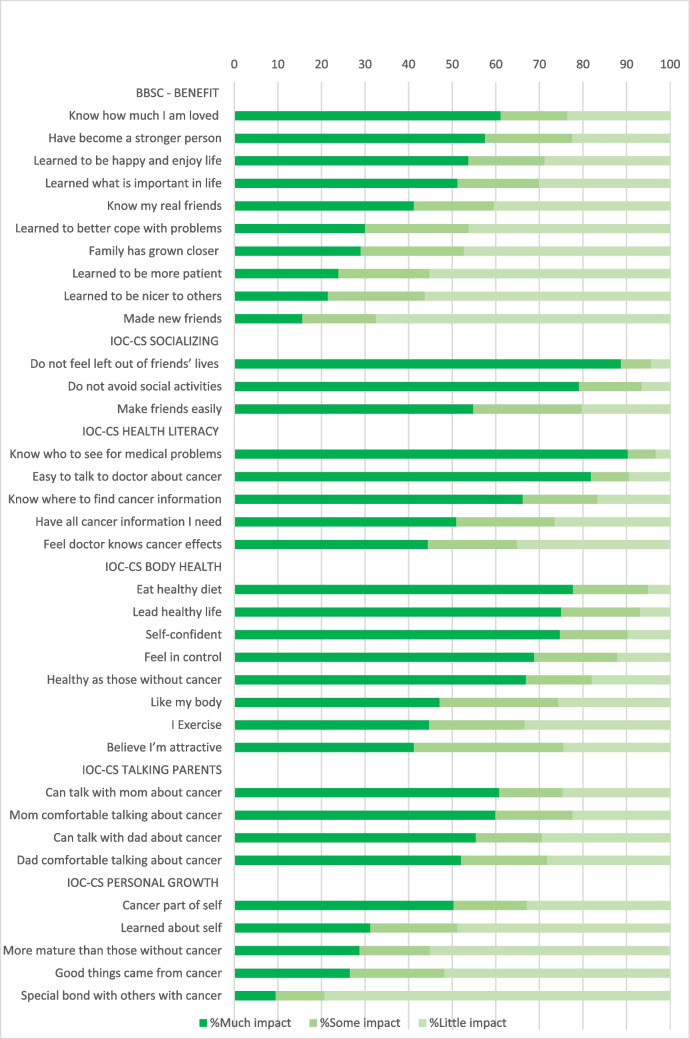
Frequencies Positive Impact of Childhood Cancer

**Fig. 3:**
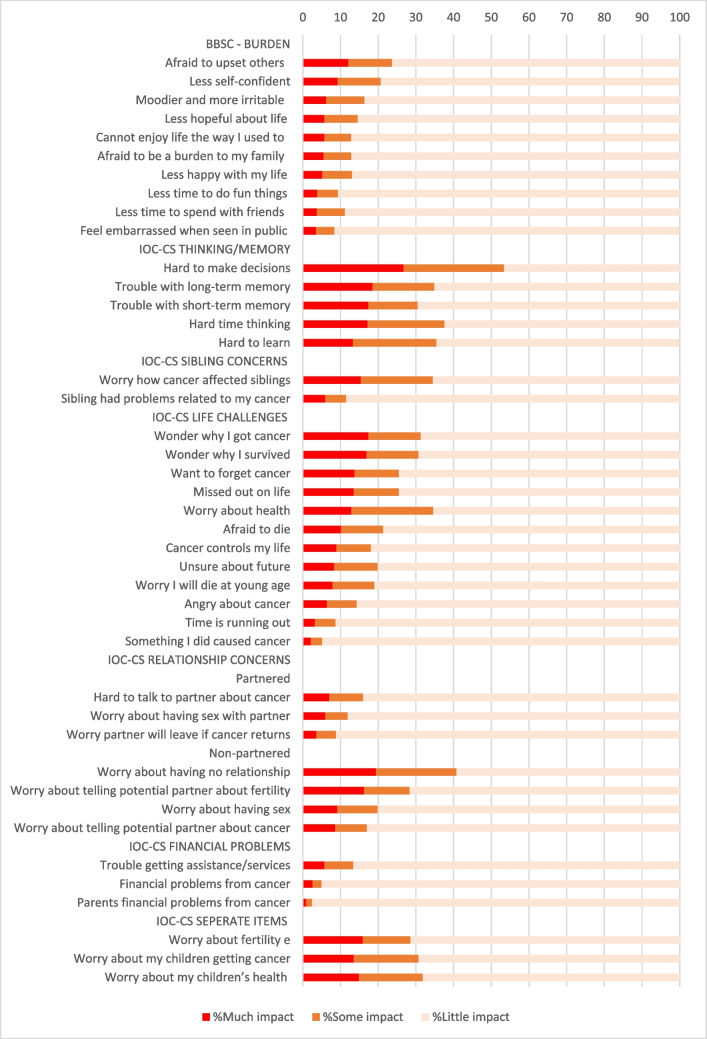
Frequencies Negative Impact of Childhood Cancer

On average, CCS reported ‘somewhat’ Benefit because of childhood cancer (M = 2.9; Supplementary Table 1). On 4 out of the 10 items, the majority (51.2–61.1%) of CCS reported much Benefit (Fig. 2). On average, CCS reported ‘not at all’ to ‘a little’ burden (M = 1.5; Supplementary Table 1) because of childhood cancer. A minority (3.5%-12.1%) reported much Burden on the individual items (Fig. 3).

#### IOC-CS

Average scores on the positive impact scales ranged from 2.5 (Personal growth) to 4.1 (Socializing), indicating ‘somewhat’ to ‘much’ impact (Supplementary Table 1). On 17 out of 25 items, the majority (50.9% -90.3%) reported much positive impact (Fig. 2). Average scores on the negative impact scales ranged from 1.4 (Financial problems) to 2.4 (Thinking/Memory), indicating ‘not at all’ to ‘somewhat’ negative impact (Supplementary Table 1). On item level, the minority reported much negative impact (2.1–26.7%) (Fig. 3).

#### Associated factors: socio-demographic and medical characteristics

Supplementary Tables 2 and 3 present the multiple linear regression models for respectively the positive and negative impact of cancer with diagnosis included in the models; Supplementary Tables 4 and 5 with treatment included. Associations were of very small to moderate size.

#### Socio-demographic characteristics

Female sex was associated with less positive impact on Body & Health, while it was associated with more Personal Growth in the model with treatment. Also, female sex was associated with more negative impact on most scales. Higher educational attainment was associated with less Benefit, but with more positive impact on Talking with Parents and Body & Health. Higher educational attainment was also associated with less Burden, and with less negative impact on Thinking/Memory and Life Challenges. Having a relationship was associated with more positive impact on Socializing and Body & Health, and with less Burden and less concerns about relationships. Being employed was associated with more positive impact on Socializing and Body & Health as well as with less Burden and with less negative impact on Thinking/Memory, Life Challenges, and Financial Problems.

#### Medical characteristics

Older age at diagnosis (both 6–11 and 12–17 vs 0–5) was associated with more Benefit and more Personal Growth, while it was also associated with more Burden in the model including treatment. Compared to CCS aged 0–5 at diagnosis, CCS aged 6–11 at diagnosis experienced more negative impact on Sibling Concerns, and CCS aged 12–17 at diagnosis more Life Challenges, both only in the model including treatment. Recurrence of cancer was associated with more Benefit, and with more Sibling Concerns in the models including diagnosis.

A CNS tumor diagnosis was associated with less positive impact on Socializing and Body & Health, and also with more Burden and more negative impact on Thinking/Memory, and Financial Problems. An unspecified/other malignancies diagnosis was associated with less Benefit, and a neuroblastoma diagnosis with less Personal Growth.

Treatment with chemotherapy was associated with more Personal Growth, but also with more negative impact on Sibling Concerns. Head/cranium radiotherapy was associated with more Benefit, and more positive impact on Health Literacy and Personal Growth, but also with more Burden and more negative impact on Thinking/Memory. Total body radiation was associated with more positive impact on Health Literacy, and testes radiotherapy with more Personal Growth.

## Discussion

This study examines positive and negative survivor-specific psychosocial outcomes in a large, nationwide cohort of adult CCS. Our results may indicate that overall CCS are resilient, with the majority of CCS reporting positive impact of cancer, and most reporting little negative impact. However, we also identified subsets of CCS facing a high degree of survivor-specific challenges, especially in the cognitive domain.

### Positive and negative impact of childhood cancer

In this study the majority of CCS reported much Benefit, and a minority much Burden on the BBSC. The BBSC has been previously used in a study among Dutch underage CCS who were 6 months to 3 years since end of treatment [13]. The present study found slightly lower scores on both Benefit and Burden which can be explained by the longer time since treatment. In line with the results on the BBSC, the majority of CCS reported impact on IOC-CS domains representing positive impact (Socializing, Talking with Parents, Body & Health, Health Literacy and Personal Growth), and a minority reported impact on domains representing negative impact (Thinking/Memory problems, Sibling Concerns, Life Challenges, Relationship Concerns, and Financial Problems). This finding is in line with previous studies [5, 12], and may indicate that CCS actually experience growth following their cancer experience or it may reflect a coping strategy in which positive impact is emphasized and negative impact denied.

The majority of CCS (> 50%) experienced much Benefit of childhood cancer, such as knowing how much they are loved, becoming a stronger person, having learned how to be happy and enjoy life, and about what is important in life. These aspects are characteristics of personal growth [32] and have been previously reported in CCS [16–19]. Surprisingly, relatively low impact was reported on the Personal Growth scale, which seems to be caused by the item ‘’I have a special bond with others with cancer’’ with only 10% of CCS experiencing this to a high degree. A study among Dutch young adult CCS also found less positive impact on this item [14]. This indicates that the growth CCS experience following their childhood cancer does not necessarily involve a special bond with others with cancer that persists long after diagnosis. Similar to literature on generic psychosocial outcomes [7], we found most CCS reporting little negative impact of childhood cancer, while subsets of CCS reported a high degree (quite a bit/ very much) of survivor-specific challenges.

Apart from the cognitive domain, an area of concern known in CCS [29, 33, 34], noticeable (> 15%) challenges among CCS were several worries they have to cope with, such as worries about how cancer affected their siblings, and wondering why they had gotten cancer and why they had survived. These worries might indicate that some CCS feel guilt after surviving cancer, which can be directed towards themselves, their siblings or to others who have not survived childhood cancer. Survivor guilt is common among people who have lived through events in which others have died [35], and attributes of survivor guilt have been reported in the cancer survivorship population, although few articles have directly addressed it [36–38]. CCS experiencing guilt and other psychosocial difficulties may benefit from cognitive behavioural therapy interventions targeting guilt and other drivers of negative mood, such as self-compassion interventions and Acceptance and Commitment Therapy [37]. Overall, > 15% of CCS worried a lot about the possibility of being infertile, especially among CCS aged 18–30 years these worries were common. Among CCS without a partner we saw worries about having no relationship and telling their potential partner about fertility. Worries about fertility were reported in other studies as well [20, 39, 40], which is not surprising as infertility is a side effect of some cancer treatments. A recent study showed the need for information regarding fertility in young adult CCS and stressed the importance of providing CCS with age-appropriate information as early in the cancer trajectory as possible and reasonable [41]. Item scores on Health Literacy of the IOC-CS in our study indeed showed that almost half of CCS do not have all cancer information they need, conforming the need for information provision even into survivorship care. This can be done for example by providing (psycho)education via websites. In line with previous studies on survivor-specific outcomes [14, 25], little negative impact was experienced on the domain of Financial Problems. Other studies, however, have reported financial hardship in CCS [42–44]. These studies have shown CCS having difficulties with affording health care, obtaining insurance coverage, and having high medical expenses. In The Netherlands these difficulties may be less prominent as basic health insurance is compulsory and insurance companies are not allowed to refuse CCS for it.

Taken together, we can conclude that CCS can experience positive impact from childhood cancer, while subgroups also experience survivor-specific challenges. Health care practitioners should acknowledge and discuss both these aspects during follow-up consultation. Targeted interventions addressing these survivor-specific challenges are important as survivor-specific outcomes are associated with psychosocial well-being [11, 14]. It is plausible that survivor-specific worries when left unaddressed eventually lead to more severe psychological problems e.g. anxiety or depression. Attention for these challenges during follow-up consultations and providing CCS with timely psychosocial support and psycho-education may prevent more severe psychological problems on the long run.

### Associated factors: socio-demographic and medical characteristics

Several socio-demographic characteristics (higher education, employment, and being in a relationship) were associated with higher positive and lower negative impact of cancer. Female CCS reported more negative impact of cancer while also experiencing greater personal growth. Previous studies found similar results [11, 12, 25].

Regarding cancer-related medical characteristics, we found CCS older at diagnosis to experience higher benefit, which has been previously reported in smaller studies [13, 15, 18]. Older children and adolescents are cognitively better equipped than younger children for mental processes such as abstract thinking and meaning making which are necessary for personal growth to occur [24]. Furthermore, we confirm previous research demonstrating that survivors of CNS tumors [11, 45, 46] and those who received head/cranium radiotherapy [45–47] experience higher negative impact, most prominent in the cognitive domain. This makes screening for cognitive deficits especially important for these subgroups. Additionally, we found that survivors of CNS tumors experienced less positive impact on Socializing and Body & Health. This is in line with literature showing that CNS survivors more often experience social difficulties [29, 48, 49], and have poorer physical functioning and lifestyle behaviors [29, 50]. For CNS tumor survivors interventions to improve social functioning may be beneficial as well as providing information on healthy life style behaviors [51–53].

### Considerations and limitations

This is the first large cohort study in which positive and negative survivor-specific outcomes of childhood cancer are explored in detail together with their associated socio-demographic and medical factors. This is crucial to obtain a complete and nuanced picture of the consequences of the cancer experience. Nevertheless, some considerations should be taken into account with the interpretation of the results. First, the BBSC and IOC-CS measure a wide range of survivor-specific outcomes including worries about health. However, there are still challenges not explicitly included in these measures, such as fear of recurrence and fear of late effects which have been reported in the literature [21, 22, 39]. Future studies may look further into these specific health-related worries. Second, the low explained variance of the models and the overall modest associations between the associated factors and the survivor-specific psychosocial outcomes indicate that additional factors play a more important role in explaining survivor-specific psychosocial outcomes. Literature points to factors such as the presence of physical late effects, dispositional optimism, and social support [22, 24, 54]. Future research could follow a biopsychosocial approach [55] and take a range of physical, psychological and social factors into account when explaining survivor-specific psychosocial outcomes. Third, the BBSC was originally developed for children with cancer [27] and has been validated only in underage CCS [13]. However, the content of the questionnaire corresponds with benefit and burden the way these concepts have been described among adult CCS [16–19, 39]. A fourth consideration lies in the cross-sectional nature of this study that allowed us to measure survivor-specific outcomes at a single moment in time which may not be representative of a larger timeframe. Also, we could therefore not distinguish between cause and effect within the identified associations. However, we could still detect among which groups of CCS survivor-specific challenges are more common and who thus may need extra attention. Fifth, the internal consistency of two IOC-CS scales, namely Sibling Concerns and Relationship Concerns for partnered CCS, was moderate in our population of adult CCS. As internal consistency gives an indication of random error and not of systematic error, it is acceptable to use the scales for descriptive statistics, although large random errors make it more difficult to detect differences between groups [56]. Finally, there were some differences between participants and non-participants, but as differences were small and associations with psychosocial outcomes not strong, bias is unlikely.

## Conclusion

The majority of CCS reported positive impact of cancer while most CCS reported little negative impact, which may indicate resiliency in most CCS. Subsets of CCS, reported a high degree of survivor-specific challenges, especially in the cognitive domain. These challenges were more prevalent among those with certain socio-demographic characteristics (female sex, lower education, un-partnered, unemployed), CNS tumor survivors and those who had received head/cranium radiotherapy. Health care providers should be aware that, although most CCS experience little negative impact of childhood cancer, they can also experience survivor-specific challenges that warrant monitoring and screening, information provision and psychosocial support.

## Supplementary Information

Below is the link to the electronic supplementary material.

Supplementary file1 (DOCX 70.9 KB)

## References

[1] Howlader N, Noone AM, Krapcho M, Miller D, Brest A, Yu M, Ruhl J, Tatalovich Z, Mariotto A, Lewis DR, Chen HS, Feuer EJ, Cronin KA (eds). SEER Cancer Statistics Review, 1975-2018, National Cancer Institute. Bethesda, MD. https://seer.cancer.gov/csr/1975_2018/, based on November 2020 SEER data submission, posted to the SEER web site, April 2021.

[2] Bitsko MJ, et al. Psychosocial Late Effects in Pediatric Cancer Survivors: A Report From the Children’s Oncology Group. Pediatr Blood Cancer. 2016;63(2):337–43. 26488337 10.1002/pbc.25773 PMC4715481

[3] Zeltzer LK, et al. Psychological status in childhood cancer survivors: a report from the Childhood Cancer Survivor Study. J Clin Oncol. 2009;27(14):2396–404. 19255309 10.1200/JCO.2008.21.1433 PMC2677925

[4] Rueegg CS, et al. Health-related quality of life in survivors of childhood cancer: the role of chronic health problems. J Cancer Surviv. 2013;7(4):511–22. 23784593 10.1007/s11764-013-0288-4

[5] van Erp LME, et al. Health-related quality of life in Dutch adult survivors of childhood cancer: A nation-wide cohort study. Eur J Cancer. 2021;152:204–14. 34119924 10.1016/j.ejca.2021.04.033

[6] Michel G, et al. Psychological distress in adult survivors of childhood cancer: the Swiss Childhood Cancer Survivor study. J Clin Oncol. 2010;28(10):1740–8. 20194864 10.1200/JCO.2009.23.4534

[7] Maas A, Maurice-Stam H, Kremer LCM, van der Aa-van Delden A, van Dulmen-den Broeder E, Tissing WJE, Loonen JJ, van der Pal HJH, de Vries ACH, van den Heuvel-Eibrink MM, Ronckers C, Neggers S, Bresters D, Louwerens M, van der Heiden-van der Loo M, van Gorp M, Grootenhuis M; Dutch LATER study group. Psychosocial outcomes in long-term Dutch adult survivors of childhood cancer: the DCCSS-LATER 2 psychooncology study. Cancer. 2023. 10.1002/cncr.34795.

[8] Jim HS, Jacobsen PB. Posttraumatic stress and posttraumatic growth in cancer survivorship: a review. Cancer J. 2008;14(6):414–9. 19060607 10.1097/PPO.0b013e31818d8963

[9] Duran B. Posttraumatic growth as experienced by childhood cancer survivors and their families: a narrative synthesis of qualitative and quantitative research. J Pediatr Oncol Nurs. 2013;30(4):179–97. 23657991 10.1177/1043454213487433

[10] Weinstein AG, et al. Roles of positive psychological outcomes in future health perception and mental health problems: A report from the Childhood Cancer Survivor Study. Psychooncology. 2018;27(12):2754–60. 30189119 10.1002/pon.4881 PMC6452629

[11] Zebrack BJ, Landier W. The perceived impact of cancer on quality of life for post-treatment survivors of childhood cancer. Qual Life Res. 2011;20(10):1595–608. 21452086 10.1007/s11136-011-9893-8

[12] Zebrack BJ, et al. Perceived positive impact of cancer among long-term survivors of childhood cancer: a report from the childhood cancer survivor study. Psychooncology. 2012;21(6):630–9. 21425388 10.1002/pon.1959 PMC3697081

[13] Maurice-Stam H, et al. Measuring perceived benefit and disease-related burden in young cancer survivors: validation of the Benefit and Burden Scale for Children (BBSC) in The Netherlands. Support Care Cancer. 2011;19(8):1249–53. 21667049 10.1007/s00520-011-1206-9 PMC3128272

[14] van Erp LME, et al. A vulnerable age group: the impact of cancer on the psychosocial well-being of young adult childhood cancer survivors. Support Care Cancer. 2021;29(8):4751–61. 33527229 10.1007/s00520-021-06009-y PMC8236461

[15] Sleurs C, Musoro J, Rowsell A, Kicinski M, Suciu S, Chantziara S, Coens C, Pe M, Missotten P,Vandecruys E, Uyttebroeck A, Dresse MF, Pluchart C, Ferster A, Freycon C, van der Werff Ten Bosch J, Rohrlich PS, Benoit Y, Darlington AS, Piette C. Sociodemographic and medical determinants of quality of life in long-term childhood acute lymphoblastic leukemia survivors enrolled in EORTC CLG studies. Cancers (Basel). 2021;14(1):152. 10.3390/cancers14010152. 10.3390/cancers14010152 PMC8750449 35008314

[16] Gunst DC, Kaatsch P, Goldbeck L. Seeing the good in the bad: which factors are associated with posttraumatic growth in long-term survivors of adolescent cancer? Support Care Cancer. 2016;24(11):4607–15. 27349524 10.1007/s00520-016-3303-2

[17] Barakat LP, Alderfer MA, Kazak AE. Posttraumatic growth in adolescent survivors of cancer and their mothers and fathers. J Pediatr Psychol. 2006;31(4):413–9. 16093518 10.1093/jpepsy/jsj058

[18] Yi J, et al. Posttraumatic Growth Outcomes and Their Correlates Among Young Adult Survivors of Childhood Cancer. J Pediatr Psychol. 2015;40(9):981–91. 26286227 10.1093/jpepsy/jsv075 PMC4707188

[19] Gianinazzi ME, et al. Cancer’s positive flip side: posttraumatic growth after childhood cancer. Support Care Cancer. 2016;24(1):195–203. 26003421 10.1007/s00520-015-2746-1

[20] Langeveld NE, et al. Quality of life, self-esteem and worries in young adult survivors of childhood cancer. Psychooncology. 2004;13(12):867–81. 15386796 10.1002/pon.800

[21] Wroot H, et al. Fear of cancer recurrence among survivors of childhood cancer. Psychooncology. 2020;29(7):1132–40. 32281171 10.1002/pon.5387

[22] Deimling GT, et al. Cancer-related health worries and psychological distress among older adult, long-term cancer survivors. Psychooncology. 2006;15(4):306–20. 16041841 10.1002/pon.955

[23] McDonnell GA, et al. The relationship between cancer-related worry and posttraumatic growth in adolescent and young adult cancer survivors. Psychooncology. 2018;27(9):2155–64. 29843190 10.1002/pon.4785 PMC6156934

[24] Turner JK, Hutchinson A, Wilson C. Correlates of post-traumatic growth following childhood and adolescent cancer: A systematic review and meta-analysis. Psychooncology. 2018;27(4):1100–9. 29096418 10.1002/pon.4577

[25] Zebrack BJ, et al. Psychometric evaluation of the Impact of Cancer (IOC-CS) scale for young adult survivors of childhood cancer. Qual Life Res. 2010;19(2):207–18. 20058086 10.1007/s11136-009-9576-x PMC2906664

[26] Feijen EAM, Teepen JC, van Dulmen-den Broeder E, van den Heuvel-Eibrink MM, van der Heidenvan der Loo M, van der Pal HJH, de Vries ACH, Louwerens M, Bresters D, Versluys B, de Ridder H, Veening M, van Leeuwen FE, Grootenhuis M, Maurice-Stam H, van Santen HM, Neggers SJCMM, Pluijm S, den Hartogh J, Ronckers CM, Tissing WJE, Loonen JJ, Kremer LCM. Clinical evaluation of late outcomes in Dutch childhood cancer survivors: methodology of the DCCSS LATER 2 study. Pediatr Blood Cancer. 2023;70(5):e30212. 10.1002/pbc.30212. 10.1002/pbc.30212 36651687

[27] Phipps S, Long AM, Ogden J. Benefit Finding Scale for Children: preliminary findings from a childhood cancer population. J Pediatr Psychol. 2007;32(10):1264–71. 17210581 10.1093/jpepsy/jsl052

[28] Currier JM, Hermes S, Phipps S. Brief report: Children’s response to serious illness: perceptions of benefit and burden in a pediatric cancer population. J Pediatr Psychol. 2009;34(10):1129–34. 19342537 10.1093/jpepsy/jsp021 PMC2782252

[29] van Gorp M, et al. Increased health-related quality of life impairments of male and female survivors of childhood cancer: DCCSS LATER 2 psycho-oncology study. Cancer. 2022;128(5):1074–84. 34726782 10.1002/cncr.34003 PMC9298191

[30] Reulen RC, et al. Health-status of adult survivors of childhood cancer: a large-scale population-based study from the British Childhood Cancer Survivor Study. Int J Cancer. 2007;121(3):633–40. 17405119 10.1002/ijc.22658

[31] Cohen J. Statistical power analysis for the behavioral sciences. New York: Academy Press; 1988.

[32] Tedeschi RG, Calhoun LG. Posttraumatic Growth: Conceptual Foundation and Empircal Evidence. Psychol Inq. 2004;15(1):1–18.

[33] Pierson C, Waite E, Pyykkonen B. A meta-analysis of the neuropsychological effects of chemotherapy in the treatment of childhood cancer. Pediatr Blood Cancer. 2016;63(11):1998–2003. 27463220 10.1002/pbc.26117

[34] Butler RW, Haser JK. Neurocognitive effects of treatment for childhood cancer. Ment Retard Dev Disabil Res Rev. 2006;12(3):184–91. 17061287 10.1002/mrdd.20110

[35] Murray H, Pethania Y, Medin E. Survivor Guilt: A Cognitive Approach. Cogn Behav Therap. 2021;14:e28. 34557258 10.1017/S1754470X21000246 PMC7611691

[36] Tetteh DA. Uncertainty and Guilt in Ovarian Cancer Survivorship. Cancer Invest. 2022;40(7):642–53. 35686723 10.1080/07357907.2022.2088779

[37] Perloff T, et al. Survivor guilt: The secret burden of lung cancer survivorship. J Psychosoc Oncol. 2019;37(5):573–85. 30798776 10.1080/07347332.2019.1569191 PMC8189655

[38] Glaser S, Knowles K, Damaskos P. Survivor guilt in cancer survivorship. Soc Work Health Care. 2019;58(8):764–75. 31311446 10.1080/00981389.2019.1640337

[39] Yi J, Kim MA, Sang J. Worries of childhood cancer survivors in young adulthood. Eur J Oncol Nurs. 2016;21:113–9. 26952686 10.1016/j.ejon.2016.02.003

[40] Newton K, et al. Facing the unknown: uncertain fertility in young adult survivors of childhood cancer. J Cancer Surviv. 2021;15(1):54–65. 32613442 10.1007/s11764-020-00910-x

[41] van Erp LME, et al. Support needs of Dutch young adult childhood cancer survivors. Support Care Cancer. 2022;30(4):3291–302. 34981198 10.1007/s00520-021-06723-7 PMC8723798

[42] Nipp RD, et al. Financial Burden in Survivors of Childhood Cancer: A Report From the Childhood Cancer Survivor Study. J Clin Oncol. 2017;35(30):3474–81. 28817372 10.1200/JCO.2016.71.7066 PMC5648170

[43] Nathan PC, et al. Financial Hardship and the Economic Effect of Childhood Cancer Survivorship. J Clin Oncol. 2018;36(21):2198–205. 29874136 10.1200/JCO.2017.76.4431

[44] Kuhlthau KA, et al. Health insurance coverage, care accessibility and affordability for adult survivors of childhood cancer: a cross-sectional study of a nationally representative database. J Cancer Surviv. 2016;10(6):964–71. 27072683 10.1007/s11764-016-0542-7 PMC8109280

[45] Wengenroth L, et al. Concentration, working speed and memory: cognitive problems in young childhood cancer survivors and their siblings. Pediatr Blood Cancer. 2015;62(5):875–82. 25645276 10.1002/pbc.25396 PMC5916869

[46] Ellenberg L, et al. Neurocognitive status in long-term survivors of childhood CNS malignancies: a report from the Childhood Cancer Survivor Study. Neuropsychology. 2009;23(6):705–17. 19899829 10.1037/a0016674 PMC2796110

[47] Kadan-Lottick NS, et al. Neurocognitive functioning in adult survivors of childhood non-central nervous system cancers. J Natl Cancer Inst. 2010;102(12):881–93. 20458059 10.1093/jnci/djq156 PMC2886093

[48] Schulte F, et al. Social adjustment in adolescent survivors of pediatric central nervous system tumors: A report from the Childhood Cancer Survivor Study. Cancer. 2018;124(17):3596–608. 30067866 10.1002/cncr.31593 PMC6191352

[49] Salley CG, et al. Social self-perception among pediatric brain tumor survivors compared with peers. J Dev Behav Pediatr. 2014;35(7):427–34. 25127341 10.1097/DBP.0000000000000077 PMC4152402

[50] Badr H, et al. Health-related quality of life, lifestyle behaviors, and intervention preferences of survivors of childhood cancer. J Cancer Surviv. 2013;7(4):523–34. 23749663 10.1007/s11764-013-0289-3 PMC3825822

[51] Barrera M, et al. A randomized control intervention trial to improve social skills and quality of life in pediatric brain tumor survivors. Psychooncology. 2018;27(1):91–8. 28124799 10.1002/pon.4385

[52] Barrera M, Schulte F. A group social skills intervention program for survivors of childhood brain tumors. J Pediatr Psychol. 2009;34(10):1108–18. 19321717 10.1093/jpepsy/jsp018

[53] Barakat LP, et al. Evaluation of a social-skills training group intervention with children treated for brain tumors: a pilot study. J Pediatr Psychol. 2003;28(5):299–307. 12808006 10.1093/jpepsy/jsg019

[54] Ishida Y, et al. Factors associated with the specific worries of childhood cancer survivors: Cross-sectional survey in Japan. Pediatr Int. 2016;58(5):331–7. 26860529 10.1111/ped.12940

[55] Engel GL. The need for a new medical model: a challenge for biomedicine. Psychodyn Psychiatry. 2012;40(3):377–96. 23002701 10.1521/pdps.2012.40.3.377

[56] Ponterotto JG, Ruckdeschel DE. An overview of coefficient alpha and a reliability matrix for estimating adequacy of internal consistency coefficients with psychological research measures. Percept Mot Skills. 2007;105(3 Pt 1):997–1014. 18229554 10.2466/pms.105.3.997-1014

