# Supplementary material for: Positive and negative survivor-specific psychosocial consequences of childhood cancer: the DCCSS-LATER 2 psycho-oncology study
Source: J Cancer Surviv. 2023 May 11;18(5):1505–16. doi: 10.1007/s11764-023-01394-1 (PMC11424676; doi:10.1007/s11764-023-01394-1)
Supplement: Supplementary file 1 — Supplementary file1 (DOCX 70.9 KB) [file 11764_2023_1394_MOESM1_ESM.docx]

**Supplementary Table 1.** Positive and negative consequences of childhood cancer according to the BBSC and IOC-CS

|  | **N** | **M^a^** | **S.D.** | **%Little impact^b^** | **%Some impact^c^** | **%Much impact^d^** |
| --- | --- | --- | --- | --- | --- | --- |
| BBSC – Benefit scale |  |  |  |  |  |  |
| **Total** | **1659** | **2.86** | **1.00** |  |  |  |
| Know how much I am loved | 1655 | 3.57 | 1.43 | 23.6 | 15.3 | 61.1 |
| Have become a stronger person | 1662 | 3.53 | 1.30 | 22.4 | 20.0 | 57.5 |
| Learned to be happy and enjoy life | 1638 | 3.36 | 1.43 | 28.8 | 17.5 | 53.7 |
| Learned what is important in life | 1643 | 3.31 | 1.39 | 30.1 | 18.7 | 51.2 |
| Know my real friends | 1644 | 2.90 | 1.55 | 40.5 | 18.4 | 41.2 |
| Learned to better cope with problems | 1651 | 2.64 | 1.39 | 46.2 | 23.8 | 30.0 |
| Family has grown closer | 1642 | 2.65 | 1.36 | 47.3 | 23.7 | 29.0 |
| Learned to be more patient | 1642 | 2.36 | 1.36 | 55.2 | 20.9 | 23.9 |
| Learned to be nicer to others | 1637 | 2.31 | 1.34 | 56.3 | 22.2 | 21.5 |
| Made new friends | 1642 | 1.98 | 1.30 | 67.5 | 16.9 | 15.6 |
| BBSC – Burden scale |  |  |  |  |  |  |
| **Total** | **1667** | **1.49** | **.63** |  |  |  |
| Afraid to upset others | 1634 | 1.79 | 1.18 | 76.3 | 11.6 | 12.1 |
| Less self-confident | 1646 | 1.72 | 1.09 | 79.2 | 11.5 | 9.2 |
| Moodier and more irritable | 1645 | 1.56 | .96 | 83.6 | 10.2 | 6.2 |
| Less hopeful about life | 1646 | 1.50 | .93 | 85.4 | 8.9 | 5.7 |
| Cannot enjoy life the way I used to | 1662 | 1.43 | .94 | 87.2 | 7.1 | 5.7 |
| Afraid to be a burden to my family | 1665 | 1.47 | .93 | 87.1 | 7.4 | 5.5 |
| Less happy with my life | 1664 | 1.47 | .90 | 86.9 | 8.0 | 5.1 |
| Less time to do fun things | 1663 | 1.33 | .82 | 90.7 | 5.5 | 3.8 |
| Less time to spend with friends | 1640 | 1.35 | .83 | 88.8 | 7.5 | 3.7 |
| Feel embarrassed when seen in public | 1667 | 1.32 | .78 | 91.5 | 4.9 | 3.5 |
| IOC-CS - Positive impact scales |  |  |  |  |  |  |
| **Socializing** | **1659** | **4.09** | **.78** |  |  |  |
| Do not feel left out of friends’ lives | 1649 | 4.58 | .84 | 4.3 | 7.0 | 88.7 |
| Do not avoid social activities | 1657 | 4.27 | 1.00 | 6.5 | 14.4 | 79.1 |
| Make friends easily | 1657 | 3.42 | 1.13 | 20.3 | 24.9 | 54.8 |
| **Health Literacy** | **1671** | **3.72** | **.76** |  |  |  |
| Know who to see for medical problems | 1662 | 4.34 | .77 | 3.3 | 6.4 | 90.3 |
| Easy to talk to doctor about cancer | 1666 | 4.16 | 1.03 | 9.4 | 8.8 | 81.8 |
| Know where to find cancer information | 1663 | 3.72 | 1.14 | 16.7 | 17.1 | 66.2 |
| Have all cancer information I need | 1665 | 3.30 | 1.20 | 26.5 | 22.6 | 50.9 |
| Feel doctor knows cancer effects | 1670 | 3.12 | 1.34 | 35.0 | 20.5 | 44.4 |
| **Body & Health** | **1676** | **3.56** | **.69** |  |  |  |
| Eat healthy diet | 1669 | 3.92 | .79 | 5.0 | 17.3 | 77.7 |
| Lead healthy life | 1672 | 3.88 | .86 | 6.9 | 18.1 | 75.0 |
| Self-confident | 1666 | 3.87 | .93 | 9.7 | 15.6 | 74.7 |
| Feel in control | 1668 | 3.72 | .98 | 12.1 | 19.1 | 68.8 |
| Healthy as those without cancer | 1664 | 3.68 | 1.21 | 18.0 | 15.1 | 66.9 |
| Like my body | 1655 | 3.20 | 1.10 | 25.7 | 27.2 | 47.1 |
| I Exercise | 1667 | 3.08 | 1.35 | 33.4 | 21.9 | 44.7 |
| Believe I’m attractive | 1672 | 3.16 | 1.07 | 24.5 | 34.3 | 41.2 |
| **Talking with Parents** | **1619** | **3.41** | **1.21** |  |  |  |
| Can talk with mom about cancer | 1597 | 3.52 | 1.36 | 24.7 | 14.5 | 60.8 |
| Mom comfortable talking about cancer | 1584 | 3.51 | 1.29 | 22.4 | 17.7 | 59.9 |
| Can talk with dad about cancer | 1555 | 3.34 | 1.40 | 29.4 | 15.2 | 55.4 |
| Dad comfortable talking about cancer | 1537 | 3.29 | 1.35 | 28.2 | 19.8 | 52.0 |
| **Personal Growth** | **1662** | **2.50** | **.89** |  |  |  |
| Cancer part of self | 1653 | 3.19 | 1.43 | 33.0 | 16.8 | 50.3 |
| Learned about self | 1644 | 2.63 | 1.34 | 48.8 | 20.0 | 31.2 |
| More mature than those without cancer | 1663 | 2.44 | 1.46 | 55.0 | 16.2 | 28.7 |
| Good things came from cancer | 1647 | 2.52 | 1.32 | 51.7 | 21.7 | 26.5 |
| Special bond with others with cancer | 1659 | 1.75 | 1.05 | 79.3 | 11.2 | 9.5 |
| IOC-CS – Negative impact scales |  |  |  |  |  |  |
| **Thinking/Memory** | **1659** | **2.35** | **.81** |  |  |  |
| Hard to make decisions | 1653 | 2.81 | 1.16 | 46.6 | 26.7 | 26.7 |
| Trouble with long-term memory | 1655 | 2.22 | 1.26 | 65.1 | 16.4 | 18.5 |
| Trouble with short-term memory | 1655 | 2.09 | 1.26 | 69.5 | 13.1 | 17.4 |
| Hard time thinking | 1655 | 2.24 | 1.20 | 62.4 | 20.4 | 17.2 |
| Hard to learn | 1651 | 2.38 | .99 | 64.4 | 22.2 | 13.3 |
| **Sibling Concerns** | **1556** | **1.74** | **.89** |  |  |  |
| Worry how cancer affected siblings | 1560 | 2.11 | 1.20 | 65.5 | 19.1 | 15.4 |
| Sibling had problems related to my cancer | 1562 | 1.38 | .91 | 88.5 | 5.6 | 5.9 |
| **Life Challenges** | **1663** | **1.73** | **.66** |  |  |  |
| Wonder why I got cancer | 1660 | 2.06 | 1.31 | 68.7 | 13.9 | 17.4 |
| Wonder why I survived | 1661 | 2.03 | 1.29 | 69.4 | 13.8 | 16.9 |
| Want to forget cancer | 1658 | 1.85 | 1.21 | 74.5 | 11.8 | 13.7 |
| Missed out on life | 1648 | 1.86 | 1.23 | 74.6 | 12.0 | 13.5 |
| Worry about health | 1665 | 2.19 | 1.09 | 65.3 | 21.7 | 12.9 |
| Afraid to die | 1658 | 1.76 | 1.14 | 78.6 | 11.2 | 10.1 |
| Cancer controls my life | 1661 | 1.64 | 1.06 | 81.9 | 9.2 | 8.9 |
| Unsure about future | 1656 | 1.69 | 1.06 | 80.1 | 11.6 | 8.3 |
| Worry I will die at young age | 1661 | 1.68 | 1.06 | 81.0 | 11.1 | 7.9 |
| Angry about cancer | 1661 | 1.50 | .98 | 85.7 | 7.9 | 6.4 |
| Time is running out | 1661 | 1.31 | .76 | 91.3 | 5.5 | 3.2 |
| Something I did caused cancer | 1661 | 1.18 | .61 | 94.9 | 3.0 | 2.1 |
| **Relationship Concerns total** | **1652** | **1.61** | **.73** |  |  |  |
| **Partnered** | **1186** | **1.50** | **.64** |  |  |  |
| Hard to talk to partner about cancer | 1181 | 1.77 | .94 | 84.0 | 9.0 | 7.0 |
| Worry about having sex with partner | 1183 | 1.43 | .94 | 88.1 | 5.9 | 6.0 |
| Worry partner will leave if cancer returns | 1180 | 1.31 | .77 | 91.2 | 5.2 | 3.6 |
| **Non-partnered** | **466** | **1.89** | **.86** |  |  |  |
| Worry about having no relationship | 468 | 2.26 | 1.27 | 59.2 | 21.4 | 19.4 |
| Worry about telling potential partner about fertility | 463 | 1.98 | 1.28 | 71.7 | 12.1 | 16.2 |
| Worry about having sex | 462 | 1.68 | 1.08 | 80.1 | 10.8 | 9.1 |
| Worry about telling potential partner about cancer | 466 | 1.62 | 1.03 | 83.0 | 8.4 | 8.6 |
| **Financial Problems** | **1605** | **1.24** | **.52** |  |  |  |
| Trouble getting assistance/services | 1604 | 1.44 | .95 | 86.5 | 7.7 | 5.7 |
| Financial problems from cancer | 1598 | 1.18 | .66 | 95.0 | 2.4 | 2.6 |
| Parents financial problems from cancer | 1587 | 1.10 | .43 | 97.5 | 1.6 | 0.9 |
| **Separate items not represented in scales**  Worry about fertility ^e^  Worry about my children getting cancer  Worry about my children’s health | 1639  1635  1630 | 1.94  2.01  2.07 | 1.32  1.20  1.22 | 71.4  69.3  68.2 | 12.7  17.2  16.9 | 15.9  13.5  14.9 |

^a^Item scores: 1 = “none at all”, 2 = “a little bit” 3 = “somewhat”, 4 = “quite a bit” 5 = “very much’’. Scale scores are presented as mean item score

BBSC = Benefit and Burden Scale Children, IOC-CS = Impact of Cancer – Childhood Survivor

^b^ Little impact = Not at all (1) or a little bit (2)

^c^ Some impact = Somewhat (3)

^d^ Much impact = Quite a bit (4) or very much (5)

**^e^** Worries about fertility in the age group 18-30 years: 52.0% little impact, 23.1% somewhat impact, 24.9% much impact.

| **Supplementary Table 2.**  Multiple Linear Regression Models with Socio-Demographic and Medical Factors including Diagnosis for Each Psychosocial Outcome – Positive Impact Scales | | | | | | |
| --- | --- | --- | --- | --- | --- | --- |
|  | **Benefit**  **BBSC** | **Socializing**  **IOC-CS** | **Talking with Parents IOC-CS** | **Body & Health**  **IOC-CS** | **Health Literacy**  **IOC-CS** | **Personal Growth**  **IOC-CS** |
| *N* | 1488 | 1491 | 1454 | 1503 | 1501 | 1493 |
|  | *β (95% CI)* | *β (95% CI)* | *β (95% CI)* | *β (95% CI)* | *β (95% CI)* | *β (95% CI)* |
| **Socio-demographic characteristics** |  |  |  |  |  |  |
| Sex (ref: male) | .137  (.036; .237) | -.061  (-.159; .037) | -.100  (-.205; .004) | **-.227****  (-.323; -.130) | -.179**  (-.281; -.077) | .180**  (.078; .282) |
| Attained age (continuous) | .028  (-.029; .084) | *-.096***  (-.151; -.041) | *-.129***  (-.188; -.069) | -.071  (-.125; -.016) | -.027  (-.084; .031) | -.015  (-.072; .043) |
| Level of education (ref: low) |  |  |  |  |  |  |
| Middle | -.161  (-.324; .002) | .021  (-.138; .180) | **.262***  (.091; .432) | .166  (.010; .322) | .028  (-.137; .194) | .005  (-.160; .170) |
| High | **-.365****  (-.526; -.203) | .117  (-.040; .274) | **.316****  (.147; .485) | **.397****  (.242; .552) | -.066  (-.230; .098) | .045  (-.119; .209) |
| Relationship (ref: no) | -.023  (-.148; .101) | **.343****  (.223; .463) | .032  (-.096; .159) | **.221****  (.103; .339) | .051  (-.074; .177) | -.049  (-.174; .076) |
| Employment (ref: no) | .018  (-.129; .165) | **.379****  (.235; .523) | .082  (-.070; .235) | **.417****  (.272; .558) | .180  (.030; .330) | -.070  (-.219; .080) |
| **Medical characteristics** |  |  |  |  |  |  |
| Age at diagnosis (years, ref 0-5) |  |  |  |  |  |  |
| 6-11 | **.321****  (.192; .4­­­49) | -.074  (-.200; .051) | .042  (-.091; .174) | .038  (-.085; .161) | -.039  (-.170; .091) | **.335****  (.205; .465) |
| 12-17 | **.335****  (.180; .489) | -.043  (-.193; .107) | .064  (-.096; .225) | -.028  (-.176; .119) | -.005  (-.162; .152) | **.334****  (.178; .490) |
| Recurrence (any vs none) | **.298****  (.151; .445) | .051  (-.093; .196) | .079  (-.074; .233) | -.123  (-.264; .019) | .162  (.011; .312) | .190  (.040; .341) |
| Diagnosis (ref: leukemias) |  |  |  |  |  |  |
| Lymphomas | -.147  (-.292; -.002) | .062  (-.080; .204) | -.204  (-.354; -.054) | -.064  (-.203; .075) | -.042  (-.190; .106) | -.119  (-.267; .028) |
| CNS tumors | -.050  (-.249; .148) | **-.395****  (-.589; -.200) | -.139  (-.344; .066) | **-.311***  (-.502; -.120) | .032  (-.171; .236) | -.053  (-.255; .149) |
| Neuroblastoma | -.274  (-.501; -.047) | .092  (-.128; .312) | -.042  (-.278; .193) | -.093  (-.310; .124) | .032  (-.199; .263) | **-.333***  (-.563; -.104) |
| Retinoblastoma | -.396  (-1.120; .327) | -.712  (-1.418; -.007) | .648  (-.093; 1.388) | -.513  (-1.210; .183) | -.018  (-.756; .720) | -.153  (-.886; .581) |
| Renal tumors | -.240  (-.411; -.068) | .076  (-.091; .244) | .172  (-.005; .350) | -.071  (-.236; .093) | .070  (-.104; .244) | -.120  (-.293; .054) |
| Hepatic tumors | -.380  (-.896; .136) | -.190  (-.677; .296) | .072  (-.439; .582) | -.173  (-.653; .307) | -.173  (-.682; .336) | -.340  (-.846; .166) |
| Bone tumors | -.031  (-.247; .185) | .028  (-.183; .238) | -.187  (-.411; .038) | -.142  (-.349; .066) | .195  (-.025; .415) | .100  (-.119; .319) |
| Soft-tissue sarcomas | -.174  (-.381; .033) | -.140  (-.340; .060) | -.026  (-.242; .190) | -.169  (-.366; .028) | -.212  (-.421; -.003) | .038  (-.170; .246) |
| Germ cell tumors | -.023  (-.313; .268) | -.049  (-.330; .231) | -.110  (-.416; .195) | -.042  (-.318; .235) | .036  (-.258; .329) | .023  (-.272; .317) |
| Unspecified and other malignancies | **-.645****  (-1.039; -.252) | .113  (-.271; .497) | -.169  (-.579; .242) | -.038  (-.417; .341) | -.542  (-.944; -.141) | -.349  (-.749; .050) |
| *R²*  *F* | .079  **6.616**** | .079  **6.681**** | .048  **3.772**** | .101  **8.777**** | .027  **2.185*** | .055  **4.520**** |

*Note.* *p-value <.004 **p-value<.001, significant results (p <.004) with standardized regression coefficient *β ≥ 0.2* are presented in bold.

| **Supplementary Table 3.**  Multiple Linear Regression Models with Socio-Demographic and Medical Factors including Diagnosis for Each Psychosocial Outcome – Negative Impact Scales | | | | | | |
| --- | --- | --- | --- | --- | --- | --- |
|  | **Burden**  **BBSC** | **Thinking/Memory**  **IOC-CS** | **Sibling Concerns**  **IOC-CS** | **Life Challenges**  **IOC-CS** | **Relationship Concerns**  **IOC-CS** | **Financial Problems**  **IOC-CS** |
| *N* | 1495 | 1491 | 1405 | 1494 | 1487 | 1442 |
|  | *β (95% CI)* | *β (95% CI)* | *β (95% CI)* | *β (95% CI)* | *β (95% CI)* | *β (95% CI)* |
| **Socio-demographic characteristics** |  |  |  |  |  |  |
| Sex (ref: male) | **.238****  (.143; .333) | **.310****  (.212; .408) | **.313****  (.207; .418) | **.219****  (.118; .320) | .177**  (.081; .273) | .106  (.003; .210) |
| Attained age (continuous) | *.102***  (.048; .156) | .053  (-.002; .108) | -.027  (-.087; .032) | .041  (-.016; .097) | -.001  (-.055; .053) | .082  (.024; .140) |
| Level of education (ref: low) |  |  |  |  |  |  |
| Middle | **-.290****  (-.443; -.137) | -.216  (-.375; -.057) | -.107  (-.280; .066) | **-.256***  (-.419; -.093) | -.123  (-.280; .033) | -.053  (-.223; .116) |
| High | **-.425****  (-.577; -.273) | **-.511****  (-.668; -.353) | -.071  (-.242; .100) | **-.372****  (-.534; -.210) | -.094  (-.249; .062) | -.228  (-.395; -.060) |
| Relationship (ref: no) | **-.251****  (-.369; -.134) | -.146  (-.267; -.026) | .000  (-.131; .130) | -.176  (-.300; -.052) | **-.448****  (-.566; -.329) | -.031  (-.158; .095) |
| Employment (ref: no) | **-.395****  (-.534; -.256) | **-.284****  (-.429; -.140) | -.097  (-.254; .060) | **-.310****  (-.457; -.162) | -.119  (-.260; .022) | **-.287****  (-.440; -.135) |
| **Medical characteristics** |  |  |  |  |  |  |
| Age at diagnosis (years, ref 0-5) |  |  |  |  |  |  |
| 6-11 | .196*  (.075; .318) | .042  (-.083; .168) | .193*  (.059; .328) | .138  (.010; .267) | .002  (-.120; .125) | .027  (-.105; .158) |
| 12-17 | .111  (-.035; .257) | -.045  (-.196; .105) | .021  (-.141; .182) | .184  (.030; .338) | .000  (-.148; .147) | .055  (-.103; .214) |
| Recurrence (any vs none) | .146  (.006; .286) | .000  (-.145; .144) | **.239***  (.084; .393) | .050  (-.098; .199) | -.030  (-.172; .111) | .065  (-.089; .218) |
| Diagnosis (ref: leukemias) |  |  |  |  |  |  |
| Lymphomas | -.011  (-.148; .127) | -.112  (-.245; .030) | -.059  (-.212; .094) | -.074  (-.220; .072) | -.065  (-.204; .074) | .025  (-.125; .174) |
| CNS tumors | **.518****  (.330; .706) | **.358****  (.163; .553) | -.221  (-.429; -.014) | -.009  (-.209; .190) | .075  (-.116; .265) | **.304***  (.100; .507) |
| Neuroblastoma | -.138  (-.354; .077) | -.164  (-.384; .057) | -.184  (-.424; .056) | -.161  (-.388; .066) | -.161  (-.377; .056) | -.144  (-.381; .093) |
| Retinoblastoma | -.062  (-.748; .624) | -.057  (-.764; .650) | -.406  (-1.146; .334) | -.325  (-.1050; .400) | .160  (-.531; .851) | -.152  (-.885; .580) |
| Renal tumors | -.115  (-.277; .046) | -.116  (-.284; .052) | -.148  (-.330; .035) | -.063  (-.235; .108) | -.041  (-.205; .123) | -.105  (-.280; .070) |
| Hepatic tumors | -.147  (-.619; .326) | -.266  (-.754; .221) | -.501  (-1.029; .026) | -.099  (-.599; .401) | .213  (-.264; .689) | -.269  (-.774; .236) |
| Bone tumors | .104  (-.100; .308) | -.020  (-.231; .191) | .247  (.022; .473) | -.052  (-.269; .164) | .010  (-.196; .216) | .028  (-.193; .248) |
| Soft-tissue sarcomas | .137  (-.058; .332) | -.016  (-.218; .186) | .052  (-.165; .269) | .149  (-.056;.355) | .038  (-.159; .236) | .087  (-.126; .300) |
| Germ cell tumors | .120  (-.152; .393) | .112  (-.172; .396) | -.112  (-.410; .185) | .284  (-.007; .575) | -.023  (-.298; .251) | -.223  (-.525; .080) |
| Unspecified and other malignancies | -.317  (-.690; .056) | -.198  (-.582; .187) | -.245  (-.648; .159) | .170  (-.224; .565) | -.152  (-.528; .224) | .050  (-.356; .456) |
| *R²*  *F* | .138  **12.479**** | .109  **9.456**** | .055  **4.225**** | .070  **5.865**** | .055  **4.524**** | .049  **3.850**** |

*Note.* *p-value <.004 **p-value<.001, significant results (p <.004) with standardized regression coefficient *β ≥ 0.2* are presented in bold.

| **Supplementary Table 4.** Multiple Linear Regression Models with Socio-Demographic and Medical Factors including Treatment for Each Psychosocial Outcome – Positive Impact Scales | | | | | | |
| --- | --- | --- | --- | --- | --- | --- |
|  | **Benefit**  **BBSC** | **Socializing**  **IOC-CS** | **Talking with Parents IOC-CS** | **Body & Health**  **IOC-CS** | **Health Literacy**  **IOC-CS** | **Personal Growth**  **IOC-CS** |
| *N* | 1483 | 1486 | 1450 | 1498 | 1496 | 1488 |
|  | *β (95% CI)* | *β (95% CI)* | *β (95% CI)* | *β (95% CI)* | *β (95% CI)* | *β (95% CI)* |
| **Socio-demographic characteristics** | | | | | | |
| Sex (ref: male) | *.156**  (.057; .256) | -.053  (-.151; .045) | -.084  (-.188; .019) | **-.216****  (-.312; -.120) | *-.153**  (-.254; -.052) | **.201****  (.100; .301) |
| Attained age (continuous) | -.019  (-.080; .042) | -.060  (-.120;.000) | *-.138***  (-.202; -.073) | -.053  (-.111; .006) | -.058  (-.120; .003) | -.038  (-.100; .023) |
| Level of education (ref: low) |  |  |  |  |  |  |
| Middle | -.149  (-.312; .014) | .037  (-.124; .198) | **.286***  (.115; .458) | .164  (.007; .321) | .060  (-.106; .225) | .019  (-.146; .184) |
| High | **-.333****  (-.495; -.170) | .123  (-.037; .284) | **.358****  (.186; .529) | **.394****  (.237; .550) | -.008  (-.173; .157) | .070  (-.094; .234) |
| Relationship (ref: no) | .014  (-.111; .139) | **.345****  (.223; .466) | .056  (-.073; .185) | **.214****  (.095; .333) | .097  (-.029; .222) | -.030  (-.155; .095) |
| Employment (ref: no) | .023  (-.124; .171) | **.368****  (.222; .515) | .087  (-.067; .241) | **.411****  (.269; .553) | .191  (.041; .341) | -.062  (-.211; .088) |
| **Medical characteristics** | | | | | | |
| Age at diagnosis (years, ref 0-5) |  |  |  |  |  |  |
| 6-11 | **.347****  (.227; .467) | -.093  (-.212; .025) | -.070  (-.195; .055) | .031  (-.085; .146) | -.076  (-.198; .046) | **.364****  (.243; .486) |
| 12-17 | **.411****  (.269; .554) | -.084  (-.225; .057) | -.051  (-.201; .098) | -.056  (-.193; .082) | -.011  (-.156; .134) | **.414****  (.270; .558) |
| Recurrence (any vs none) | .169  (.009; .329) | .036  (-.123; .195) | -.019  (-.187; .148) | -.103  (-.257; .052) | -.040  (-.203; .124) | .047  (-.116; .210) |
| Treatment |  |  |  |  |  |  |
| Surgery (ref: no) | -.053  (-.163; .056) | -.043  (-.151; .064) | .120  (.006; .233) | -.061  (-.166; .044) | .125  (.014; .236) | .029  (-.082; .139) |
| Chemotherapy (ref: no) | .230  (.067; .392) | .103  (-.056; .262) | .131  (-.038; .301) | .090  (-.066; .246) | .231  (.066; .397) | .**294****  (.129; .458) |
| Radiotherapy area (ref: no) |  |  |  |  |  |  |
| Head/ cranium | **.334****  (.175; .492) | -.180  (-.336; -.024) | .228  (.062; .394) | -.074  (-.226; .079) | **.289****  (.128; .450) | **.289****  (.128; .449) |
| Spinal | .071  (-.197; .339) | -.030  (-.296; .236) | -.023  (-.304; .258) | -.206  (-.465; .053) | .121  (-.152; .394) | -.127  (-.398; .144) |
| Total body irradiation | .143  (-.123; .408) | .027  (-.237; .291) | .251  (-.024; .526) | -.068  (-.324; .189) | **.498****  (.228; .769) | .251  (-.019; .522) |
| Thorax | -.021  (-.240; .197) | -.094  (-.308; .120) | -.084  (-.311; .142) | -.084  (-.294; .126) | .160  (-.062; .381) | -.021  (-.241; .199) |
| Abdominal/ pelvic | .110  (-.082; .302) | -.036  (-.226; .154) | -.072  (-.276; .131) | -.107  (-.293; .078) | -.043  (-.238; .152) | .030  (-.164; .224) |
| Testes | .696  (.003; 1.388) | .416  (-.267; 1.099) | .112  (-.651; .876) | .578  (-.092; 1.247) | .483  (-.223; 1.189) | **1.289****  (.589; 1.990) |
| Neck | -.003  (-.272; .267) | .144  (-.122; .409) | .014  (-.268; .296) | .081  (-.179; .341) | -.037  (-.311; .238) | -.082  (-.354; .190) |
| Upper extremities | .361  (-.215; .937) | .383  (-.162; .927) | .457  (-.164; 1.079) | -.387  (-.920; .147) | .175  (-.387; .738) | -.241  (-.799; .317) |
| Lower extremities | -.010  (-.430; .410) | .096  (-.318; .511) | -.317  (-.759; .126) | .173  (-.233; .579) | .198  (-.230; .626) | .292  (-.133; .716) |
| Radioisotopes | .232  (-.301; .766) | .010  (-.517; .536) | .302  (-.248; .852) | .086  (-.429; .602) | .075  (-.469; .618) | .414  (-.126; .953) |
| *R²*  *F* | .090  **6.896**** | .071  **5.363**** | .048  **3.465**** | .104  **8.156**** | .040  **2.954**** | .073  **5.523**** |

*Note.* *p-value <.004 **p-value<.001, significant results (p <.004) with standardized regression coefficient *β ≥ 0.2* are presented in bold.

| **Supplementary Table 5.** Multiple Linear Regression Models with Socio-Demographic and Medical Factors including Treatment for Each Psychosocial Outcome – Negative Impact Scales | | | | | | |
| --- | --- | --- | --- | --- | --- | --- |
|  | **Burden**  **BBSC** | **Thinking/Memory**  **IOC-CS** | **Sibling Concerns**  **IOC-CS** | **Life challenges**  **IOC-CS** | **Relationship Concerns**  **IOC-CS** | **Financial Problems**  **IOC-CS** |
| *N* | 1490 | 1486 | 1401 | 1489 | 1482 | 1437 |
|  | *β (95% CI)* | *β (95% CI)* | *β (95% CI)* | *β (95% CI)* | *β (95% CI)* | *β (95% CI)* |
| **Socio-demographic characteristics** | | | | | | |
| Sex (ref: male) | **.233****  (.138; .329) | **.313****  (.215; .411) | **.303****  (.197; .408) | **.227****  (.127; .327) | .168**  (.072; .264) | .098  (-.006; .201) |
| Attained age (continuous) | .052  (-.006; .110) | .008  (-.051; .068) | -.034  (-.099; .030) | .026  (-.036; .087) | -.020  (-.078; .039) | .053  (-.010; .116) |
| Level of education (ref: low) |  |  |  |  |  |  |
| Middle | **-.291****  (-.446; -.135) | -.223  (-.384; -.063) | -.096  (-.271; .078) | -.233  (-.397; -.068) | -.140  (-.297; .018) | -.066  (-.238; .105) |
| High | **-.420****  (-.575; -.265) | **-.502****  (-.662; -.342) | -.052  (-.225; .122) | **-.367****  (-.531; -.203) | -.122  (-.279; .035) | -.229  (-.400; -.059) |
| Relationship (ref: no) | **-.238****  (-.357; -.118) | -.130  (-.252; -.008) | .021  (-.110; .153) | -.166  (-.290; -.041) | **-.445****  (-.564; -.326) | -.036  (-.164; .091) |
| Employment (ref: no) | **-.387****  (-.528; -.246) | **-.275****  (-.420; -.130) | -.081  (-.239; .078) | **-.306****  (-.455; -.158) | -.138  (-.280; .005) | **-.285****  (-.439; -.130) |
| **Medical characteristics** | | | | | | |
| Age at diagnosis (years, ref 0-5) |  |  |  |  |  |  |
| 6-11 | **.266****  (.152; .381) | .074  (-.044; .192) | .**231****  (.104; .359) | .155  (.034; .276) | .028  (-.087; .143) | .067  (-.058; .192) |
| 12-17 | **.245****  (.108; .382) | .036  (-.104; .176) | .099  (-.052; .250) | **.255****  (.111; .398) | .047  (-.090; .184) | .128  (-.020; .276) |
| Recurrence (any vs none) | .119  (-.035; .273) | -.043  (-.201; .115) | .201  (.030; .371) | .014  (-.148; .177) | .005  (-.149; .160) | .012  (-.157; .181) |
| Treatment |  |  |  |  |  |  |
| Surgery (ref: no) | .037  (-.068; .142) | .014  (-.093; .121) | .016  (-.099; .132) | .028  (-.082; .138) | -.022  (-,127; .083) | -.009  (-.123; .104) |
| Chemotherapy (ref: no) | .009  (-.147; .164) | -.119  (-.278; .041) | **.310****  (.139; .481) | .117  (-.047; .281) | .020  (-.135; .175) | -.086  (-.255; .083) |
| Radiotherapy area (ref: no) |  |  |  |  |  |  |
| Head/ cranium | **.246***  (.094; .398) | **.276****  (.120; .432) | .190  (.021; .358) | .088  (-.072; .247) | .027  (-.125; .180 | .110  (-.057; .277) |
| Spinal | .247  (-.011; .506) | .133  (-.131; .396) | -.054  (-.335; .227) | .095  (-.175; .365) | -.120  (-.378; .137) | .143  (-.139; .426) |
| Total body irradiation | .050  (-.204; .305) | .057  (-.204; .318) | .018  (-.262; .298) | .226  (-.041; .494) | .025  (-.230; .280) | .274  (-.004; .552) |
| Thorax | -.062  (-.272; .148) | -.024  (-.238; .190) | .032  (-.198; .262) | -.195  (-.414; .024) | -.046  (-.255; .164) | .074  (-.152; .301) |
| Abdominal/ pelvic | **.**185  (.001; .370) | .082  (-.107; .272) | .070  (-.133; .272) | .211  (.018; .404) | .180  (-.005; .365) | .117  (-.084; .318) |
| Testes | -.087  (-.752; .577) | -.045  (-.726; .636) | -.081  (-.838; .677) | .185  (-.513; .883) | -.168  (-.833; .496) | .173  (-.536; .882) |
| Neck | -.150  (-.409; .109) | -.225  (-.490; .040) | -.100  (-.380; .180) | .059  (-.212; .331) | -.127  (-.386; .132) | -.043  (-.324; .238) |
| Upper extremities | .195  (-.335; .725) | .129  (-.414; .672) | .265  (-.329; .860) | .294  (-.262; .851) | .147  (-.383; .676) | .226  (-.392; .845) |
| Lower extremities | -.055  (-.467; .358) | -.112  (-.526; .301) | .011  (-.423; .445) | -.166  (-.590; .257) | -.173  (-.576; .231) | -.083  (-.534; .367) |
| Radioisotopes | -.192  (-.704; .321) | .045  (-.479; .570) | .185  (-.366; .735) | -.109  (-.647; .428) | -.244  (-.755; .268) | -.154  (-.700; .391) |
| *R²*  *F* | .129  **10.336**** | .110  **8.588**** | .053  **3.653**** | .072  **5.421**** | .056  **4.126**** | .045  **3.143**** |

*Note.* *p-value <.004 **p-value<.001, significant results (p <.004) with standardized regression coefficient *β ≥ 0.2* are presented in bold.
